# Widespread Albedo Decreasing and Induced Melting of Himalayan Snow and Ice in the Early 21^st^ Century

**DOI:** 10.1371/journal.pone.0126235

**Published:** 2015-06-03

**Authors:** Jing Ming, Yaqiang Wang, Zhencai Du, Tong Zhang, Wanqin Guo, Cunde Xiao, Xiaobin Xu, Minghu Ding, Dongqi Zhang, Wen Yang

**Affiliations:** 1 National Climate Centre, China Meteorological Administration, Beijing, China; 2 State Key Laboratory of Cryospheric Sciences, Cold and Arid Regions Environmental and Engineering Research Institute, Chinese Academy of Sciences, Lanzhou, China; 3 Chinese Academy of Meteorological Sciences, Beijing, China; 4 Institute of Atmospheric Physics, Chinese Academy of Sciences, Beijing, China; 5 Interdisciplinary Mathematics Institute, University of South Carolina, Columbia, South Carolina, United States of America; 6 Chinese Research Academy of Environmental Sciences, Beijing, China; 7 Snow-Ice-Aerosol Analyzing Laboratory, Beijing, China; Peking University, CHINA

## Abstract

**Background:**

The widely distributed glaciers in the greater Himalayan region have generally experienced rapid shrinkage since the 1850s. As invaluable sources of water and because of their scarcity, these glaciers are extremely important. Beginning in the twenty-first century, new methods have been applied to measure the mass budget of these glaciers. Investigations have shown that the albedo is an important parameter that affects the melting of Himalayan glaciers.

**Methodology/Principal Findings:**

The surface albedo based on the Moderate Resolution Imaging Spectroradiometer (MODIS) data over the Hindu Kush, Karakoram and Himalaya (HKH) glaciers is surveyed in this study for the period 2000–2011. The general albedo trend shows that the glaciers have been darkening since 2000. The most rapid decrease in the surface albedo has occurred in the glacial area above 6000 m, which implies that melting will likely extend to snow accumulation areas. The mass-loss equivalent (MLE) of the HKH glacial area caused by surface shortwave radiation absorption is estimated to be 10.4 Gt yr^-1^, which may contribute to 1.2% of the global sea level rise on annual average (2003–2009).

**Conclusions/Significance:**

This work probably presents a first scene depicting the albedo variations over the whole HKH glacial area during the period 2000–2011. Most rapidly decreasing in albedo has been detected in the highest area, which deserves to be especially concerned.

## Introduction

**Fig 1:**
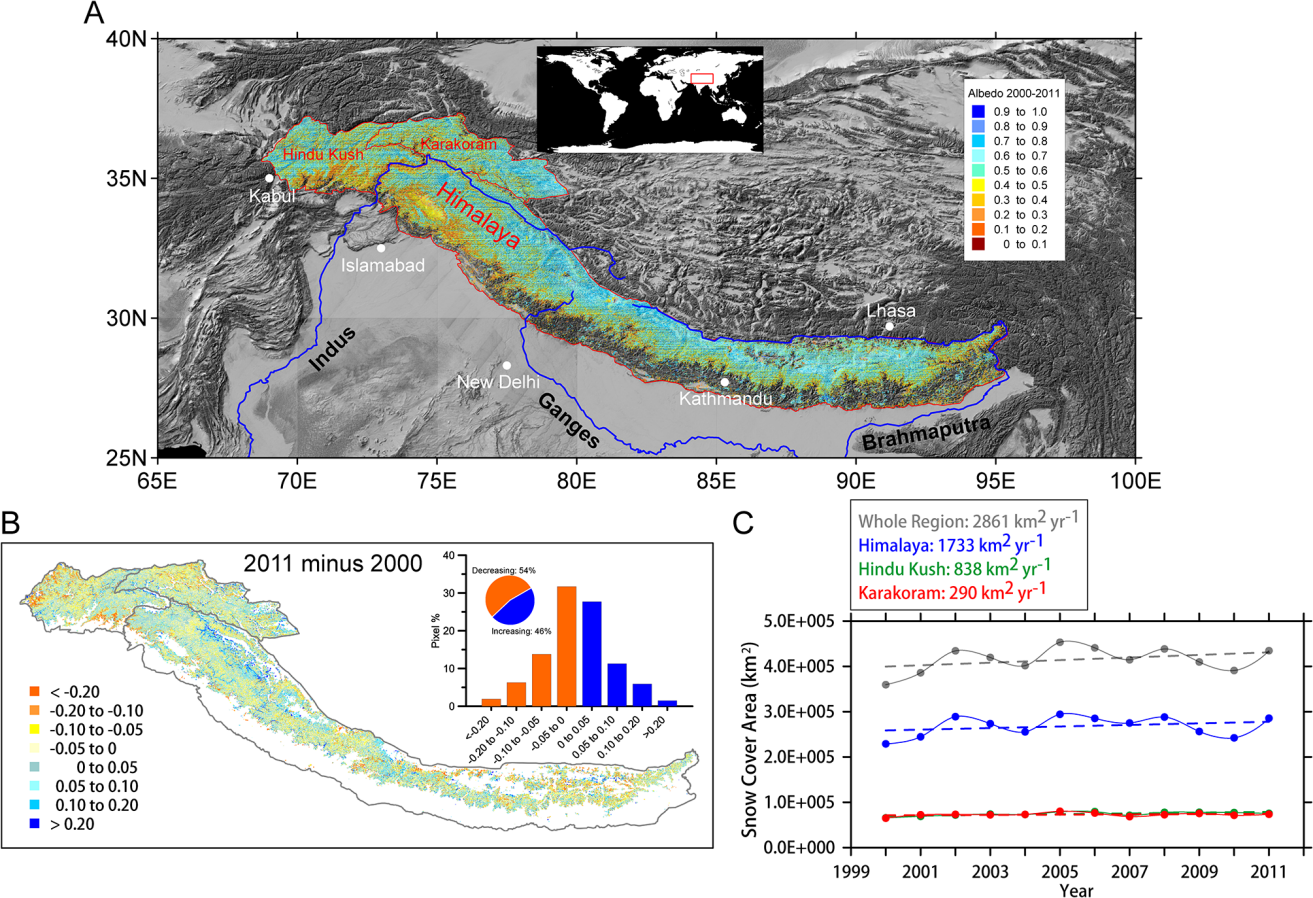
(A) The study area, which is also marked inside the red square in the global map at the top, and the overall average snow cover albedo in the Hindu Kush, Karakoram, and Himalaya regions during the period 2000–2011. (B) the difference in the snow albedos over the HKH region between 2000 and 2011; the percentages of the pixels that experience an increase or decrease and the albedo distribution are shown in the smaller plot. (C) the annual variations in snow cover in the Hindu Kush (trend at confidence level of 0.95), Karakoram (trend at confidence level of 0.55), and Himalaya regions (trend at confidence level of 0.60) and for the entire region (HKH, trend at confidence level of 0.74); the dashed lines are the linear fits.

The greater Himalaya region, which includes the Hindu Kush, Karakoram, and Himalaya (HKH) regions, has the third most abundant solid water supply on Earth following the Antarctic and Arctic. The extensive snow and glacial coverage represents a vital water resource for more than 1 billion people living along and around large international rivers, such as the Indus, Ganges, and Brahmaputra [1] (Fig 1A). The Himalaya and Karakoram regions may have lost mass due to snow and ice melt at the rate of 5 Gt yr^-1^ over the period 2003–2010 [2]. However, another report has suggested that the Himalayan glaciers experienced a much higher mass-loss equivalent (MLE) rate of 13 Gt yr^-1^ during the period 2003–2009 [3], which implies that there are disagreements regarding this topic in the literature. Nevertheless, the consensus is that the HKH region generally lost ice mass during the past decade [4], although a slight mass gain (+0.11 m water equivalent) in the Karakoram glaciers was detected between 1999 and 2008 [5].

Absorbed solar radiation is a primary energy source for heating glaciers. The mass budget of a specific glacier is related to variations in surface albedo (i.e., the ratio of reflected solar radiation to incoming solar radiation) [6]. When the albedo of a glacier decreases substantially, surface melt most likely occurs. This phenomenon has been detected in the Alps, in Greenland, and in the Himalayas [6–8]. Therefore, a complete understanding of changes in the surface albedo is essential for estimating glacial melt. Unfortunately, such information is not available for the HKH region, which limits the comprehensiveness in our understanding of albedo-related snow and glacial cover variations in the region. This work expands the study on snow and glacial albedo from some individual glaciers to the whole HKH region, presenting a probably first complete comprehension of the albedo variations of the whole HKH glaciers during the past decade, and further estimates the surface melt caused by albedo variations.

## Data and Methods

The HKH region contains vast areas of ice and snow that encompass more than 0.76 million km^2^ [9]. Ideally, proper satellite datasets should be used for investigating surface snow albedo variations over such a wide-range area. The Moderate Resolution Imaging Spectroradiometer (MODIS), which is onboard the Terra satellite, snow product (MOD10A1) is used in this study. The product contains snow albedo data with a daily temporal resolution and a 500 m spatial resolution since February 2000. The MODIS biases compared with the actual geolocations are within 50 m at nadir [10].

Compared with other satellite data, the MOD10A1 dataset has a relatively longer data record and finer resolution; therefore, this dataset is conducive for studying snow and glacial albedo variations in the HKH region over the last decade. The MOD10A1 albedo data have been validated for the Turkish mountainous glacial region [11]. The temporal trends between ground measurements and MODIS data and their average absolute differences correspond to within 10% and are much better at higher elevations [11]. Investigations in the High Asian mountainous glaciers [12] and the French Alps [6] have suggested that it is reasonable to use the MOD10A1 dataset to study the surface albedo variations and trends in mountainous glaciers within regions of complex terrain. In this study, we also use this dataset to explore the possible relationship between albedo and mass variations within the snow- and ice-covered areas of the HKH region.

The MOD10A1 dataset used in this study is the level 3 MODIS snow data product. The dataset uses a sinusoidal map projection that divides the global surface into 36 (horizontal) × 18 (vertical) tiles. Each tile covers an area of 1200 × 1200 km2 and contains 2400 × 2400 grid points, and each grid point contains daily quality assessment (QA), fractional snow cover (FSC), snow cover, and albedo data [13]. Six tiles (h23v5, h24v5, h24v6, h25v6, and h26v6) cover the entire HKH region (i.e., Hindu Kush, Karakoram, and Himalaya) (S1 Fig). These tiles can be freely downloaded from the NASA-supported web server (ftp://n4ftl011u.ecs.nasa.gov/SAN/MOST/MOD10A1.005). Approximately 1.5 × 10^11^ (i.e., 2400 columns × 2400 rows × 6 tiles × 365 days × 12 years) data points exist before further processing. The data encompass the period from February 2000 to December 2011.

The MOD10A1 daily snow cover product uses the best result from multiple daily observations that are mapped to each grid cell using a scoring algorithm; the observation nearest to nadir with the greatest coverage at the highest solar elevation angle is selected. Therefore, the possibility of mapping pixels with large off-nadir viewing angles onto the grid cell is minimized [14]. Because large amounts of data must be processed, a GIS software tool for visualizing and analyzing meteorological data is used (MeteoInfo) [15]. This tool provides technical skills. Moreover, the tool can “mine” the gridded data within a certain geographic boundary that contain non-snow grids (S1 Fig) and provide useful statistics. The required criteria for selecting the albedo data are as follows: 1) a specific grid point must contain snow cover (i.e., excluding pure bare land); 2) a grid point must be defined as “good quality” by passing the spatial QA (i.e., poor quality data are excluded, such as clouds); 3) the grid is 100% covered by snow, which excludes the grids with mixed land cover. The albedo (0–100%) for the grid points that meet these conditions is chosen from the dataset. If the grid does not match the criteria, it is set to “not valid”.

## Results and Discussions

### Overall snow-cover albedo variations over the HKH region

The albedo map for the snow and glacial cover in the HKH for the period 2000–2011 is shown in Fig 1A; the overall average albedo is 0.541 (S2 Fig). The average snow and glacier albedos are 0.54, 0.49, and 0.55 in the Himalaya, Hindu Kush, and Karakoram regions, respectively. The northern areas of the HKH region generally have higher albedos (exceeding 0.50) than the southern regions (less than 0.50) (Fig 1A). The annual mean albedo in the HKH region varies during the period 2000–2011 (S3 Fig). Lower snow-cover albedos are primarily located at the southern margin of the HKH region, which is near the low plain and populous areas; higher albedos dominate the northern region. The snow cover albedos in the eastern Karakoram and northwestern Himalaya regions are notably higher than those in other areas. Large differences in albedo occur between 2000 and 2011; moreover, 46% of the pixels exhibit increasing albedos, whereas 54% of the pixels have decreasing albedos (Fig 1B). Nearly 60% of the pixels have an albedo variation from -0.05 to +0.05, whereas 40% of the pixels exhibit much larger variations (exceeding 0.10).

The decreasing albedo primarily occurs in the Hindu Kush, Karakoram, and the middle and eastern Himalaya regions; the increasing albedo is substantial in the northwestern Himalaya region and part of the Karakoram region. The seasonal variations suggest that the highest albedo (0.60) occurs in spring and winter and the lowest albedo occurs in summer (0.47) (S2 Fig). The albedo in the HKH region is substantially lower than that found for mountainous glaciers in Turkey (0.62) [11] and for the Greenland ice sheet (> 0.80) [16]. The total area of the snow-covered pixels (those 100% covered by snow) over the HKH region increases by ~2900 km^2^ yr^-1^ during the period 2000–2011, although the significance level is 0.74; moreover, the snow-covered areas in the three sub-regions all exhibited increasing albedo trends during this period (Fig 1C).

### Glacial-area albedo variations over the HKH region related to topography

The glacial outlines in the HKH region based on the Randolph Glacier Inventory (Version 3.2) [17] are used to interpret the pure-glacier albedo data. First, three blocks (Nos. 13, 14, and 15) are selected from the nineteen first-order glacier regions. The HKH glacier outlines are subsequently recovered using the MeteoInfo tool associated with the boundary of the HKH region [18]. Topography data are obtained from the Shuttle Radar Topography Mission (SRTM) with a spatial resolution of 90 m [19]. The dataset has height and geo-location errors within 15 m and 5 m, respectively, which meet the required accuracy for this study. A total of ~1.83×10^5^ pixels exist within the perimeter of the glacial area in the HKH region, equal to ~4.57×10^4^ km^2^ which is comparable with the glacial areas of the Himalaya and Karakoram regions as estimated by Bolch et al. [18].

**Fig 2:**
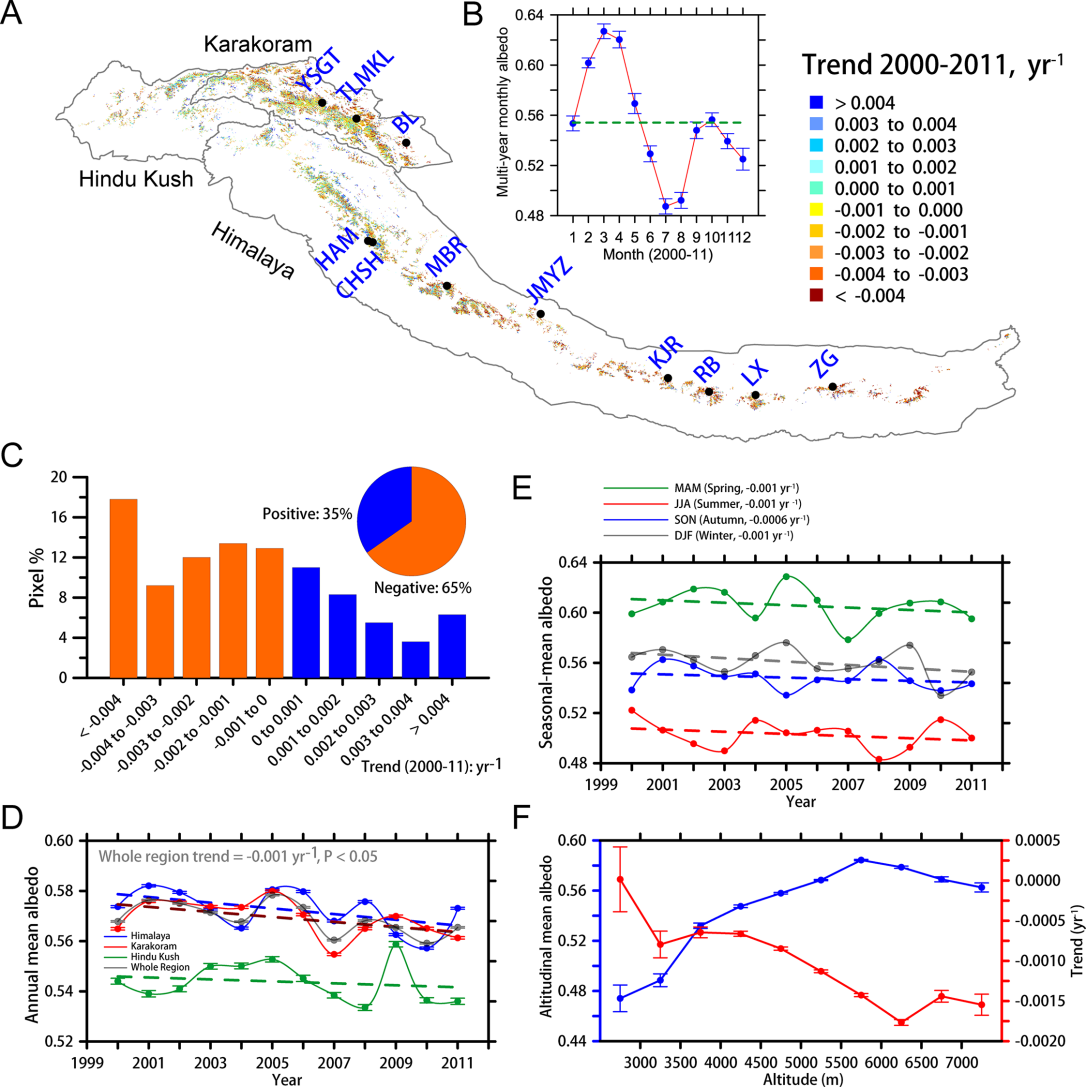
(A) Trend map of the surface albedo for the HKH glaciers (YSGT = Yinsugaiti, TLMKL = Telamukanli, BL = Bilan, MBR = Menbari, JMYZ = Jiemayangzong, KJR = Kangjiaruo, RB = Rongbuk, LX = Laxia, ZG = Zeng, CHSH = Chhota Shigri, and HAM = Hamtah; (B) the multi-year monthly mean albedo of the HKH glacial area for the period 2000–2011. The blue dots with error bars (standard error of the mean, SEM) denote the monthly mean; the green dashed line is the multi-year mean albedo. (C) The percentages of pixels with albedo trends (by category) and a pie chart of pixels with positive and negative trends. (D) The annual mean surface albedo for the HKH (grey), Hindu Kush (green), Karakoram (red), and Himalaya (blue) regions with the corresponding SEMs and trends (dashed lines). (E) Seasonal mean albedos (dots) and trends (dashed lines). (F) The average altitude-dependent albedos (blue) and trends (red) with the corresponding SEMs.

The surface albedo distribution for the HKH glaciers exhibits a similar pattern to snow cover, i.e. the albedos in eastern Karakoram and northwestern Himalaya regions are notably higher than other areas (S4 Fig). We calculate and map the linear albedo trend for each pixel in Fig 2A, which shows that the majority of pixels with negative trends are located across the entire HKH region, whereas the few with positive trends are primarily located in the northwestern Himalaya and Karakoram regions. A positive albedo trend means that more solar radiation is reflected back to space, which favors a mass gain. The trend map complies with previously reported mass losses primarily in Himalayan glaciers [4] and slight mass gains in the Karakoram glaciers [5]. The overall average albedo of the glacial area in the HKH region is approximately 0.55. The highest albedo (~0.63) occurs in March, whereas the lowest albedo (~0.49) occurs in July (Fig 2B). The typical melting season of the HKH glaciers occurs from May to October [20]; the albedo is typically below or approximately equivalent to the average albedo during this period. During the period 2000–2011, 35% of the pixels exhibit positive trends in albedo and 65% have negative trends in albedo; nearly 18% of the pixels decrease in albedo larger than 0.004 yr^-1^ (Fig 2C).

The total decrease in the surface albedo over the HKH glacial area is -0.001 yr^-1^ (95% confidence level) during the period 2000–2011. The regional trends are -0.0004 yr^-1^ for Hindu Kush, -0.001 yr^-1^ for Karakoram, and -0.001 yr^-1^ for Himalaya (Fig 2D). The trends in the HKH glacial coverage are nearly identical in spring, summer, and winter; however, the trend in autumn is slightly smaller (Fig 2E), which is a possible consequence of more frequent monsoon precipitation [21]. The albedo over the HKH glacial area exhibits strong altitudinal dependence after interpolating the terrain data onto glacial grids. The average albedo during the period 2000–2011 increases from ~0.47 below 3000 m to ~0.58 for 5500–6000 m and then decreases to 0.56 above 7000 m; the temporal trend in albedo has an inverse variation that generally decreases to more negative values with altitude (Fig 2F). Above 5500 m, which corresponds to the crest of Himalaya, the average albedo reduction exceeds -0.0015 yr^-1^ during the period 2000–2011. The most rapid reduction occurs between 6000 m and 6500 m (up to -0.0018 yr^-1^).

**Fig 3:**
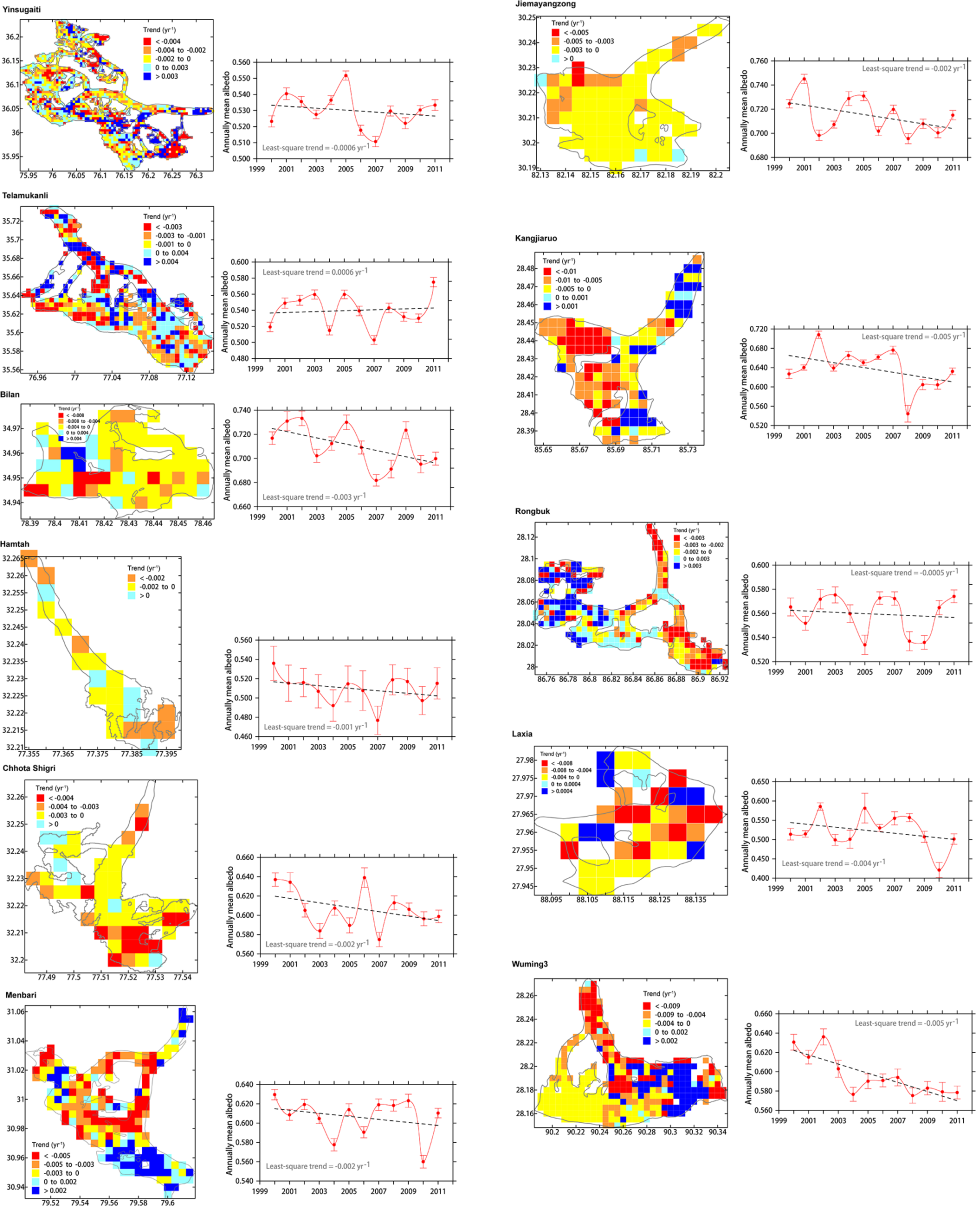
Albedo trend maps and general trends for eleven selected glaciers whose names are depicted in Fig 2. Cyan to blue colors indicate positive trends, whereas yellow to red colors indicate negative trends.

**Fig 4:**
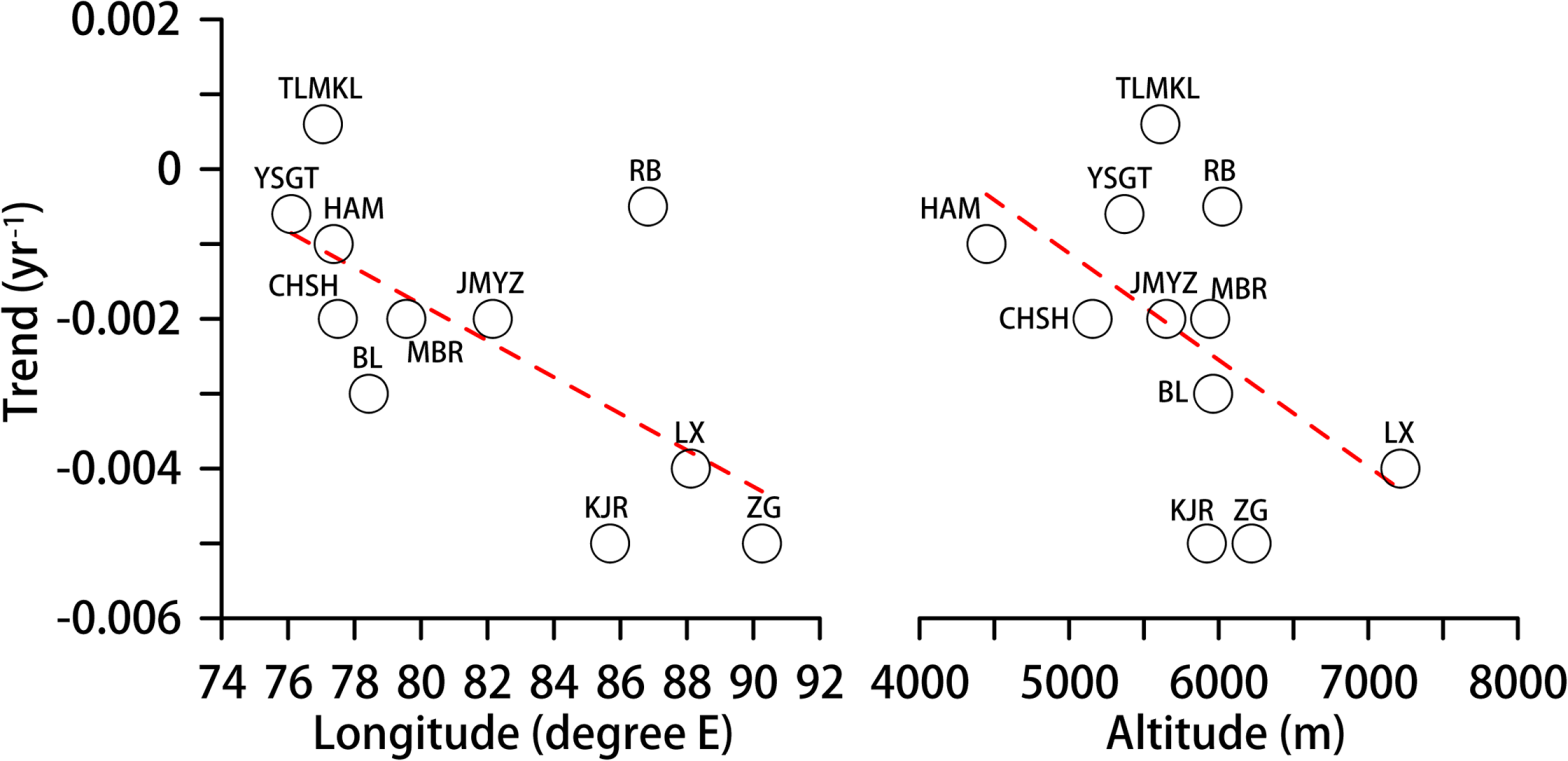
Longitude- and altitude-dependent albedo trends for the eleven selected glaciers.

To investigate the albedo variations of individual glaciers, eleven glaciers (Table 1) that are distributed across southern (CHSH and HAM) and northern slopes (the remaining nine glaciers) from the Karakoram region to the eastern Himalaya region are selected (Fig 2A), based on the quality of the mass balance records since 2000 [22]. These glaciers have areas of approximately 5 to 360 km^2^, which is estimated according to the number of pixels in each area. Ten glaciers exhibit negative surface albedo trends (-0.0005 to -0.005 yr^-1^) during the period 2000–2011, whereas TLMKL has a positive trend (0.0006 yr^-1^) in the Karakoram area (Fig 3). For all eleven glaciers, 64% and 36% of the corresponding pixels have negative and positive trends, respectively (Fig 3), which indicates that expansive surface darkening has occurred in the sampled glaciers similar to the whole HKH glaciers. The geographic dependence of the albedo in the sampled glaciers is also similar to that of the overall HKH glacial area. The surface albedo of the eastern and high glaciers is decreasing faster than that of the western and low glaciers (Fig 4).

**Table 1:** The eleven glaciers selected within the HKH region and their geographical information.

Glacier Name	Initial	Longitude (E)	Latitude (N)	Altitude (m)	Trend (yr^-1^)	Mountain Area	Pixel #
Yinsugaiti	YSGT	76.10	36.07	5370	-0.0006	Karakoram	1435
Telamukanli	TLMKL	77.05	35.63	5610	0.0006	Karakoram	445
Bilan	BL	78.43	34.96	5963	-0.003	Karakoram	75
Menbari	MBR	79.56	30.99	5943	-0.002	Himalaya	179
Jiemayangzong	JMYZ	82.16	30.21	5650	-0.002	Himalaya	74
Kangjiaruo	KJR	85.69	28.43	5921	-0.005	Himalaya	122
Rongbuk	RB	86.83	28.05	6024	-0.0005	Himalaya	280
Laxia	LX	88.12	27.96	7215	-0.004	Himalaya	36
Zeng	ZG	90.26	28.19	6220	-0.005	Himalaya	316
Chhota Shigri	CHSH	77.50	32.20	5157	-0.002	Himalaya	51
Hamtah	HAM	77.37	32.24	4448	-0.001	Himalaya	21

### Estimated surface melt detected by MODIS data and contribution to sea level rise

The energy balance for glacial melting involves longwave and shortwave radiations, sensible heat, and latent heat [23]. However, long-term observations of the mass and energy balance at three HKH glaciers, including CHSH, showed that the radiative flux is the primary driver of surface melting in summer; turbulent fluxes are only important in winter when melting is insignificant [24–26]. In this study, we presume that the radiative flux is the dominant factor that affects the surface melting of the HKH glaciers during the melt season, ignoring the influences from other energy fluxes. The timing and duration of the snowmelt over the sub-HKH regions (Table 2) were detected by the QuikSCAT satellite during the period 2000–2008 [17]. In general, the melting of the HKH snow begins in early May and ends in mid-October and exhibits little variation (Table 2).

**Table 2:** The melting durations of the Karakoram, eastern Himalaya, mid-Himalaya, and western Himalaya regions.

Sub-Region	Duration	Melting Days
Karakoram	Late May—Late September	124
Eastern Himalaya	Early May—Mid-October	161
Central Himalaya	Late May—Early October	130
Western Himalaya	Mid-May—Mid-September	124

Owing to the logistical difficulties, there are no meteorological networks in the HKH glacial areas. Only temporary monitoring of meteorological air temperature was discontinuously conducted at the ER glacier of Mt. Everest during the period 2005–2007 [27]. We use the mean temperature observed at the ER glacier during the melt season (May to October) (Table 3) and the lapse rate (6.5°C km^-1^) that is applicable at high-elevation HKH glaciers and previously proved by the meteorological station data in the western Himalaya region [28], and create a map of surface air temperature over the HKH glacial area (S5A Fig). Moreover, the relationship between the temperatures of the Himalayan snow cover and the surface air obtained from western Himalaya in-situ measurements [29] are used to estimate the surface snow temperature (T_s_). T_s_ is calculated as: T_s_ = 0.48 × T_a_ -3.3, if T_a_ < 3.6°C, or T_s_ = 0°C, if T_a_ > 3.6°C, where T_s_ is the snow surface temperature of the HKH glaciers and T_a_ is the air temperature above the snow surface during melting seasons. The snow reaches its melting point (0°C) if the air temperature above the snow exceeds 3.6°C [29], which indicates that the linear relationship is not applicable. Surface melting primarily occurs in the Hindu Kush, western Karakoram, and southern Himalaya regions (S5B Fig).

**Table 3:** The mean monthly air temperatures that were discontinuously measured at the ER glacier during the period 2005–2007.

Year	Month	Air Temperature (°C)
2005	May	-11.3
2005	June	-5.5
2005	July	-3.4
2007	October	-11.3
Average		-7.875

**Fig 5:**
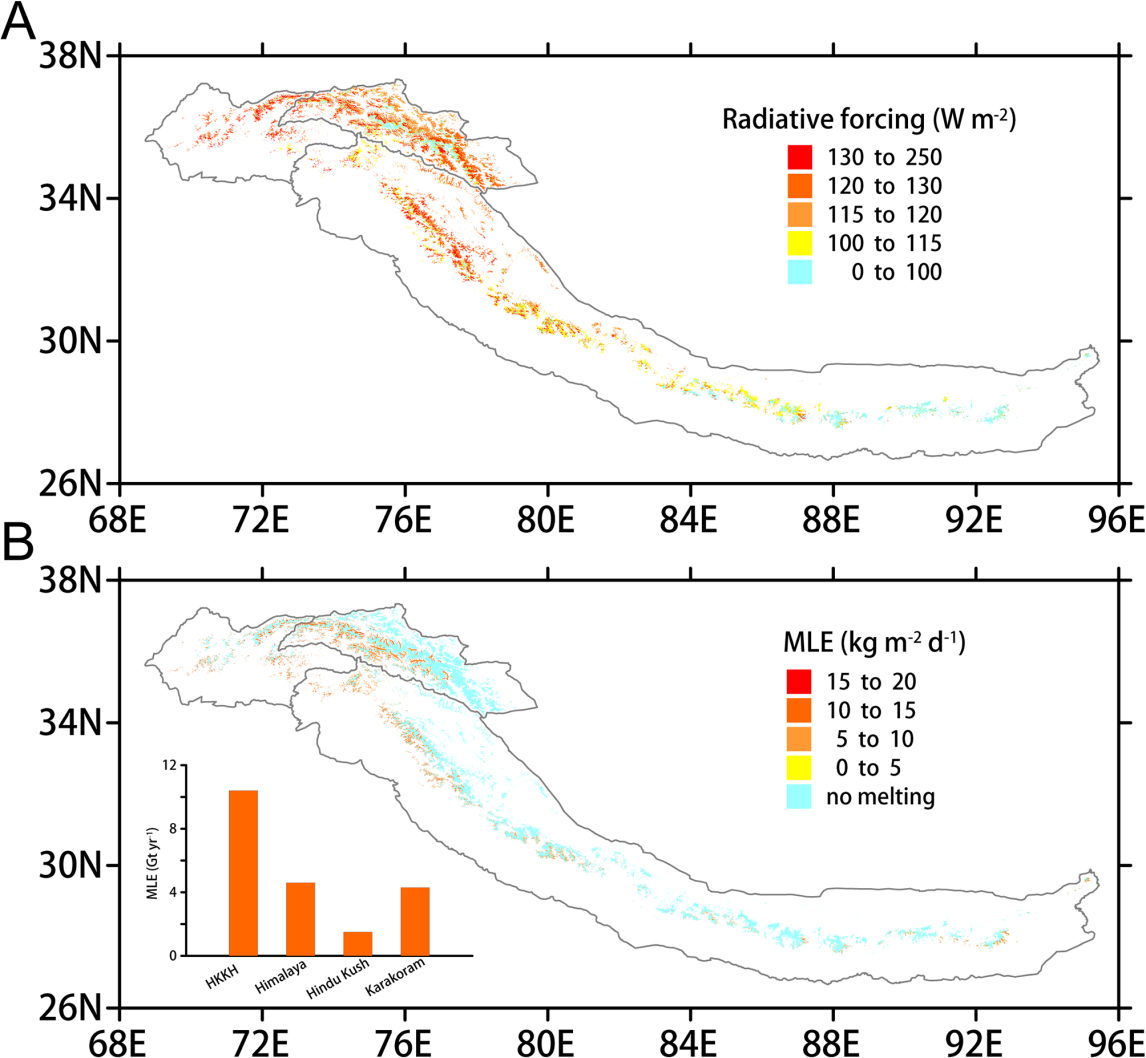
(A) The surface radiative forcing map of the HKH glaciers due to the albedo effect. (B) The daily MLE map for the HKH glacial area due to shortwave radiation absorption; the annual MLEs for the HKH, Hindu Kush, Karakoram, and Himalaya regions are also shown (column chart).

The Clouds and Earth’s Radiant Energy System (CERES) data [30] (downloadable from http://ceres.larc.nasa.gov/ with a monthly temporal resolution and a spatial resolution of 1° × 1°), are interpolated onto the finer MODIS-albedo grids for the HKH glacial area to determine the surface incoming shortwave radiation (SISR) over the HKH region and (S6 Fig). The surface radiative forcing due to the albedo effect is calculated using F = SISR × (1-α), where F (forcing) is the absorbed shortwave radiation and α is the albedo (Fig 5A). The average forcing is 116 W m^-2^ over the HKH glaciers. The forcing in the western HKH region (> 100 W m^-2^) is generally higher than that in the east (< 100 W m^-2^), which indicates greater surface energy absorption by the western glaciers. Therefore generally, the darkening trend of -0.001 yr^-1^ can result in an annual enhanced forcing of ~0.12 W m^-2^ per year in the HKH glacial area.

We calculated the daily MLE of the grid points that reach the melting point (Fig 5B) following: M = F × t / f, where M is the melted snow mass equivalent in kg m^-2^ d^-1^, F is the surface forcing in W m^-2^, t is the daily solar irradiance duration in seconds (the daily average sunshine duration in the HKH region is ~8 hours by Hua et al. [31]), and f is the latent fusion heat of snow (334000 J kg^-1^). Strong surface melting primarily occurs in the Hindu Kush, western Karakoram, and southern Himalaya regions, i.e., in areas where the daily MLE exceeds 5 kg m^-2^ during the melt season (Fig 5B). Overall, the MLE of the HKH glaciers is approximately 10.4 Gt yr^-1^ in the melt seasons of 2000–2011 due to enhanced SISR absorption, which accounts for 67% of the previously reported gross mass loss rate of 15.6 Gt yr^-1^ for the period 2003–2009 [3]. The sub-regional MLE rates are 1.5 Gt yr^-1^, 4.3 Gt yr^-1^, and 4.6 Gt yr^-1^ in the Hindu Kush, Karakoram Himalaya regions, respectively (Fig 5B). Particularly, the mass balances of the CHSH and HAM glaciers are -0.163 m w.e. yr^-1^ and -0.985 m w.e. yr^-1^ due to enhanced SISR absorption in the melt season; these values correspond to 24% and 67% of the annual mass losses for the same time period, respectively, which were previously reported by [32].

The MLE can be converted to sea level equivalent using the following equation: SLE = MW_HKH_ / S_ocean_, where SLE is the sea level equivalent in mm yr^-1^, MW_HKH_ is the MLE of the HKH glaciers in kg yr^-1^, and S_ocean_ is the global ocean area (362 × 10^12^ m^2^).The MLE rate (10.4 Gt yr^-1^) in the HKH region caused by the surface SISR absorption is equal to a SLE of ~0.03 mm per year., or to 1.2% of the observed global sea level rise rate during the period 2003–2009 [3].

## Conclusions and Perspectives

A few factors may be inducing the general darkening of the HKH glacial area, including global/regional warming [33–35] and the deposition of light-absorbing impurities on the glacial surface [36–40]. A relatively rapid decrease in the surface albedo occurs in higher glaciers; the most significant decrease is observed in the glacial area at an elevation of 6000–6500 m. The albedo decrease is related to the snow melt in the HKH region because shortwave radiation absorption at the glacial surface provides a substantial energy source to melt snow and snow melt darkens the surface; thus, a positive feedback that accelerates the melting process occurs. Some Karakoram glaciers have shown abnormal surges or advances during the past decade [5], whereas the strongest surface melting notably occurs in western Karakoram. The MLE of the glaciers in Himalaya and Karakoram are very similar. The general darkening trends do not have significant effects on the surface energy budget of the HKH glacial cover yet; a linear fit suggests that a total forcing increase of +1.2 W m^-2^ occurs from 2000 to 2011. However, the albedo decrease implies an irreversible melting scenario for the HKH glaciers in the near future.

## Supporting Information

S1 Fig (A) Study area marked with a yellow boundary; the h23v5, h24v5, h25v5, h24v6, h25v6, and h26v6 MODIS tiles are also depicted (Topography data is from the NOAA’s ETOPO1 global relief product), and (B) A sinusoidal projection of the MODIS tiles (https://lpdaac.usgs.gov/products/modis_overview). (TIF)

S2 Fig Multi-year mean monthly snow cover albedo for the HKH region; the blue dots with error bars (i.e. standard error of the mean, is standard deviation divided by the square root of the number of samples) denote the monthly mean. The green dashed line is the multi-year mean albedo.
(TIF)

S3 Fig The annual mean snow cover albedo map for the HKH region for the period 2000–2011. (TIF)

S4 Fig The annual mean glacier albedo map for the HKH region for the period 2000–2011. (TIF)

S5 Fig (A) Derived mean surface air temperatures for the melt season over the HKH glaciers based on in-situ measurements at the ER site, and (B) Mean snow surface temperature estimated from (A). (TIF)

S6 Fig Surface incoming shortwave radiation (SISR) map derived from the CERES dataset. (TIF)

## References

[1] Immerzeel W, van Beek L, Bierkens M. Climate Change Will Affect the Asian Water Towers. Science. 2010; 328: 1382–1385. 10.1126/science.1183188 20538947

[2] Jacob T, Wahr J, Pfeffer W, Swenson S. Recent contributions of glaciers and ice caps to sea level rise. Nature. 2012; 482: 514–518. 10.1038/nature10847 22318519

[3] Gardner A, Moholdt G, Cogley J, Wouters B, Arendt A, Wahr J, et al A Reconciled Estimate of Glacier Contributions to Sea Level Rise: 2003 to 2009. Science. 2013; 340: 852–857. 10.1126/science.1234532 23687045

[4] Kaab A, Berthier E, Nuth C, Gardelle J, Arnaud Y. Contrasting patterns of early twenty-first-century glacier mass change in the Himalayas. Nature. 2012; 488: 495–498. 10.1038/nature11324 22914167

[5] Gardelle J, Berthier E, Arnaud Y. Slight mass gain of Karakoram glaciers in the early twenty-first century. Nat Geosci. 2012; 5: 322–325.

[6] Dumont M, Gardelle J, Sirguey P, Guillot A, Six D, Rabatel A, et al Linking glacier annual mass balance and glacier albedo retrieved from MODIS data. The Cryosphere. 2012; 6: 1527–1539. 10.1088/0031-9155/57/6/1527 22391091

[7] Box J, Fettweis X, Stroeve J, Tedesco M, Hall D, Steffen K. Greenland ice sheet albedo feedback: thermodynamics and atmospheric drivers. The Cryosphere. 2012; 6: 821–839.

[8] Brun F, Dumont M, Wagnon P, Berthier E, Azam M, Shea JM, et al Seasonal changes in surface albedo of Himalayan glaciers from MODIS data and links with the annual mass balance. The Cryosphere. 2015; 9: 341–355.

[9] Gurung D, Giriraj A, Aung K, Shrestha B, Kulkarni A. Snow-cover mapping and monitoring in the Hindu Kush-Himalayas Kathmandu: International Centre for Integrated Mountain Development (ICIMOD); 2011.

[10] Wolfe R, Nishihama M, Fleig A, Kuyper J, Roy D, Storey J, et al Achieving sub-pixel geolocation accuracy in support of MODIS land science. Remote Sens Environ. 2002; 83: 31–49.

[11] Tekeli A, Şensoy A, Şorman A, Akyürek Z, Şorman Ü. Accuracy assessment of MODIS daily snow albedo retrievals within situ measurements in Karasu basin. Hydrol Process. 2006; 20: 705–721.

[12] Wang J, Ye B, Cui Y, He X, Yang G. Spatial and temporal variations of albedo on nine glaciers in western China from 2000 to 2011. Hydrol Process. 2014; 28: 3454–3465.

[13] Riggs G, Hall D, Salomonson V. MODIS snow products user guide to collection 5 National Snow & Ice Data Center 2006 Available: https://nsidc.org/data/docs/daac/modis_v5/dorothy_snow_doc.pdf.

[14] Huang X, Liang T, Zhang X, Guo Z. Validation of MODIS snow cover products using Landsat and ground measurements during the 2001–2005 snow seasons over northern Xinjiang, China. International Journal of Remote Sensing. 2011; 32: 133–152.

[15] Wang Y. MeteoInfo: GIS software for meteorological data visualization and analysis. Meteorological Applications. 2014; 21: 360–368.

[16] Stroeve J, Box J, Haran T. Evaluation of the MODIS (MOD10A1) daily snow albedo product over the Greenland ice sheet. Remote Sens Environ. 2006; 105: 155–171.

[17] Arendt A, Bolch T, Cogley J, Gardner A, Hagen J, Hock R, et al. Randolph Glacier Inventory—A Dataset of Global Glacier Outlines: Version 3.2. GLIMS Technical Report. 2012.

[18] Bolch T, Kulkarni A, Kaab A, Huggel C, Paul F, Cogley J, et al The state and fate of Himalayan glaciers. Science. 2012; 336: 310–314. 10.1126/science.1215828 22517852

[19] Rodriguez E, Morris C, Belz J, Chapin E, Martin J, Daffer W, et al An assessment of the SRTM topographic products JPL, NASA 2005 Available: http://www2.jpl.nasa.gov/srtm/SRTM_D31639.pdf.

[20] Panday P, Frey K, Ghimire B. Detection of the timing and duration of snowmelt in the Hindu Kush-Himalaya using QuikSCAT, 2000–2008. Environ Res Lett. 2011; 6: 024007.

[21] Palazzi E, von Hardenberg J, Provenzale A. Precipitation in the Hindu-Kush Karakoram Himalaya: Observations and future scenarios. J Geophys Res. 2013; 118: 85–100.

[22] WGMS. Fluctuations of Glaciers 2005–2010, Vol. X Zurich: World Glacier Monitoring Service; 2012.

[23] Hoffman MJ, Fountain AG, Liston GE. Surface energy balance and melt thresholds over 11 years at Taylor Glacier, Antarctica. J Geophys Res. 2008; 113: F04014.

[24] Wagnon P, Ramanathan A, Arnaud Y, Azam F, Vincent C. Long-term mass and energy balance monitoring of Himalayan glaciers (GLACIOCLIM project): some results for Chhota Shigri Glacier (India), Mera and Changri Nup glaciers (Nepal). EGU General Assembly Conference Abstracts. 2012; 14: 5328.

[25] Yang W, Guo X, Yao T, Yang K, Zhao L, Li S, et al Summertime surface energy budget and ablation modeling in the ablation zone of a maritime Tibetan glacier. J Geophys Res. 2011; 116: D14116.

[26] Azam MF, Wagnon P, Vincent C, Ramanathan AL, Favier V, Mandal A, et al Processes governing the mass balance of Chhota Shigri Glacier (western Himalaya, India) assessed by point-scale surface energy balance measurements. The Cryosphere. 2014; 8: 2195–2217.

[27] Yang X, Qun J, Liu H, Wang S. Meteorological Characteristics of the East Rongbuk Glacier, Mt. Qomolangma. Arid Meteorology (Chinese with English abstract). 2008; 26: 16–21.

[28] Jain S, Goswami A, Saraf A. Determination of land surface temperature and its lapse rate in the Satluj River basin using NOAA data. Int J Remote Sens. 2008; 29: 3091–3103.

[29] Datt P, Srivastava P, Negi P, Satyawali P. Surface energy balance of seasonal snow cover for snow-melt estimation in N-W Himalaya. J Earth Syst Sci. 2008; 117: 567–573.

[30] Kato S, Loeb N, Rose F, Doelling D, Rutan D, Caldwell TE, et al Surface Irradiances Consistent with CERES-Derived Top-of-Atmosphere Shortwave and Longwave Irradiances. J. Climate. 2013; 26: 2719–2740.

[31] Hua W, Dong Y, Fan G. The analysis of spatial and temporal characteristics of annual sunshine duration over Qinghai-Tibet Plateau. Journal of Mountain Science (Chinese with English abstract). 2010; 28: 21–30.

[32] Vincent C, Ramanathan A, Wagnon P, Dobhal D, Linda A, Berthier E, et al Balanced conditions or slight mass gain of glaciers in the Lahaul and Spiti region (northern India, Himalaya) during the nineties preceded recent mass loss. The Cryosphere. 2013; 7: 569–582.

[33] Gautam R, Hsu N, Lau K, Tsay S, Kafatos M. Enhanced pre-monsoon warming over the Himalayan-Gangetic region from 1979 to 2007. Geophys Res Lett. 2009; 36: L07704.

[34] Nie Y, Liu Q, Liu S. Glacial Lake Expansion in the Central Himalayas by Landsat Images. Plos One. 2013; 8: e83973 10.1371/journal.pone.0083973 24376778 PMC3869856

[35] Shrestha U, Gautam S, Bawa K. Widespread Climate Change in the Himalayas and Associated Changes in Local Ecosystems. Plos One. 2012; 7: e36741 10.1371/journal.pone.0036741 22615804 PMC3352921

[36] Yasunari T, Bonasoni P, Laj P, Fujita K, Vuillermoz E, Marinoni A, et al Estimated impact of black carbon deposition during pre-monsoon season from Nepal Climate Observatory—Pyramid data and snow albedo changes over Himalayan glaciers. Atmos Chem Phys. 2010; 10: 6603–6615.

[37] Nair V, Babu S, Moorthy K, Sharma A, Marinoni A, Ajai. Black carbon aerosols over the Himalayas: direct and surface albedo forcing. Tellus B. 2013; 65: 19738.

[38] Gautam R, Hsu N, Lau W, Yasunari T. Satellite observations of desert dust-induced Himalayan snow darkening. Geophys Res Lett. 2013; 40: 1–6.

[39] Painter T, Flanner M, Kaser G, Marzeion B, VanCuren R, Abdalati W. End of the Little Ice Age in the Alps forced by industrial black carbon. Proc Natl Acad Sci U.S.A. 2013; 110: 15216–15221. 10.1073/pnas.1302570110 24003138 PMC3780880

[40] Ming J, Du Z, Xiao C, Xu X, Zhang D. Darkening of the mid-Himalaya glaciers since 2000 and the potential causes. Environ Res Lett. 2012; 7: 014021.

