# Supplementary figures and images for: Widespread Albedo Decreasing and Induced Melting of Himalayan Snow and Ice in the Early 21st Century
Source: PLoS One. 2015 Jun 3;10(6):e0126235. doi: 10.1371/journal.pone.0126235 (PMC4454657; doi:10.1371/journal.pone.0126235)

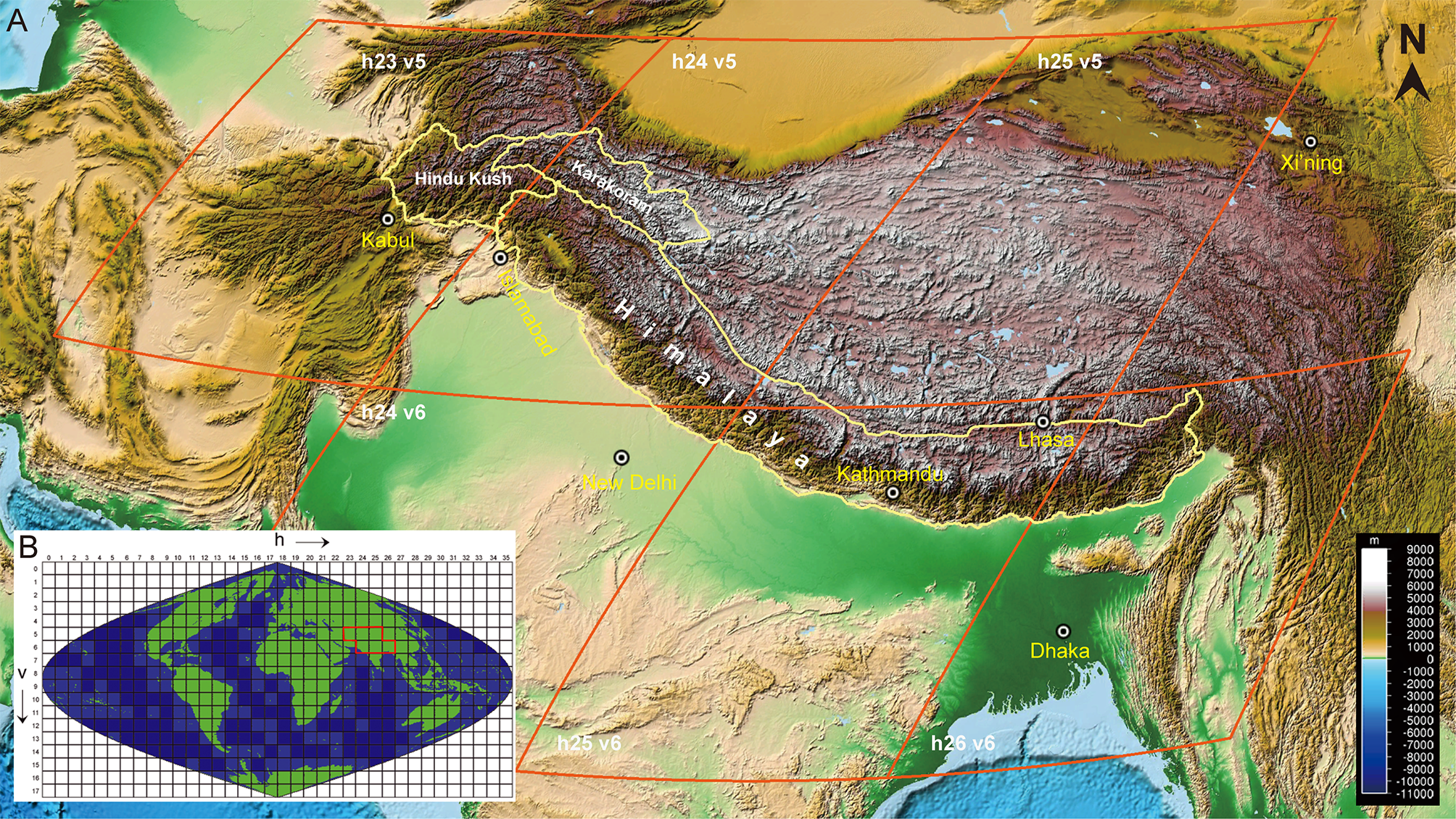

Supplement: S1 Fig — (TIF) [file pone.0126235.s001.tif]

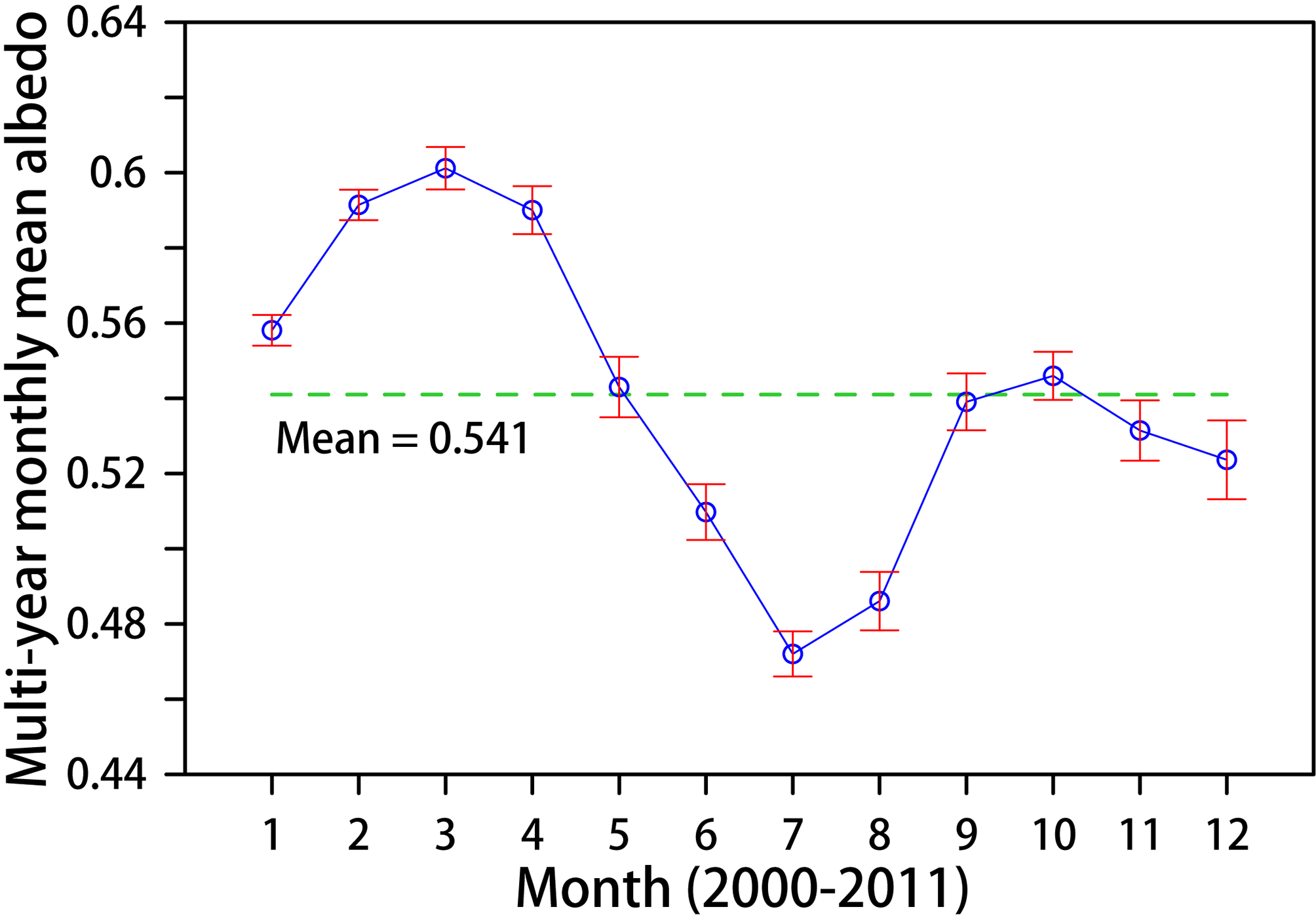

Supplement: S2 Fig — The green dashed line is the multi-year mean albedo. (TIF) [file pone.0126235.s002.tif]

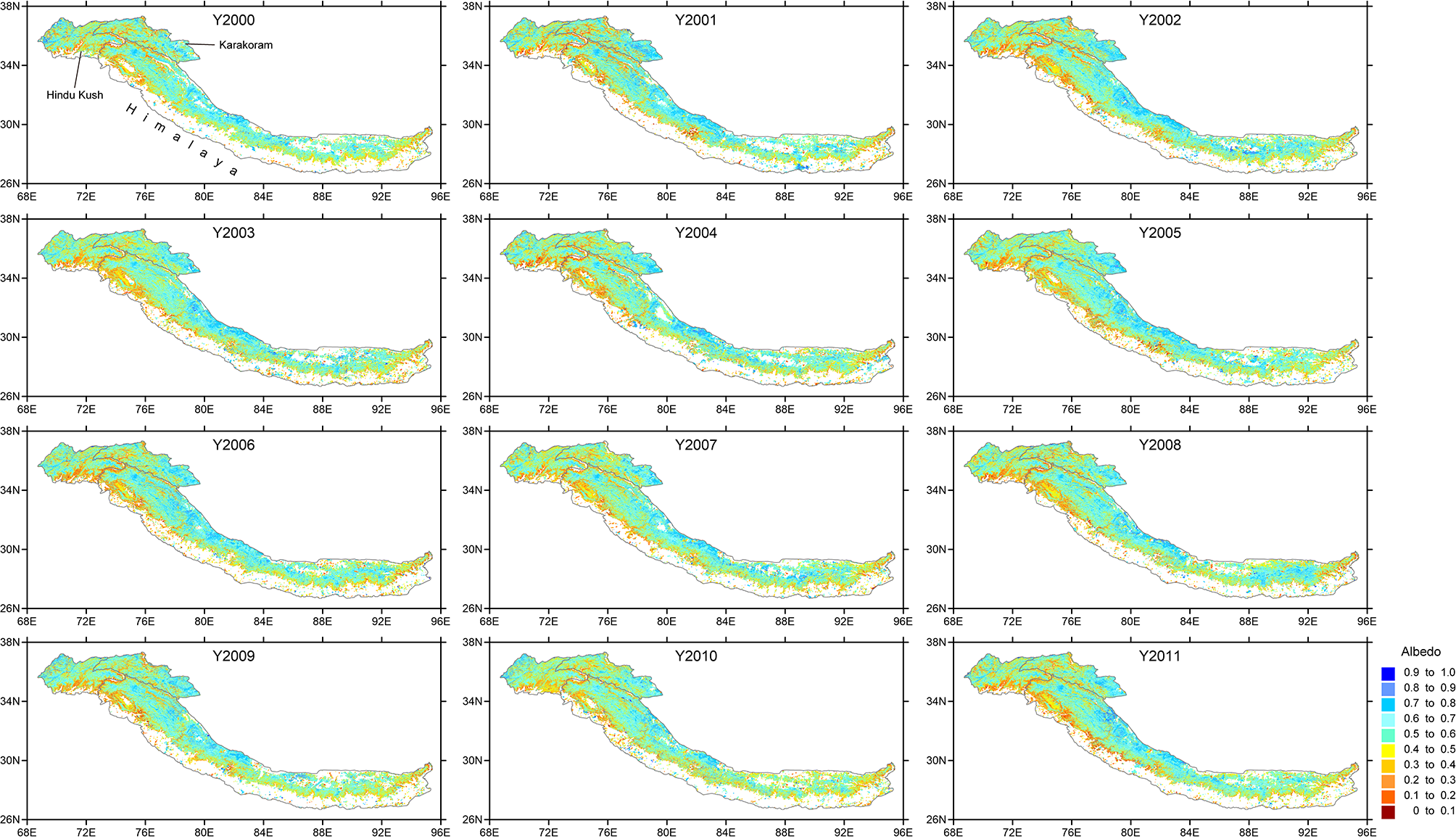

Supplement: S3 Fig — (TIF) [file pone.0126235.s003.tif]

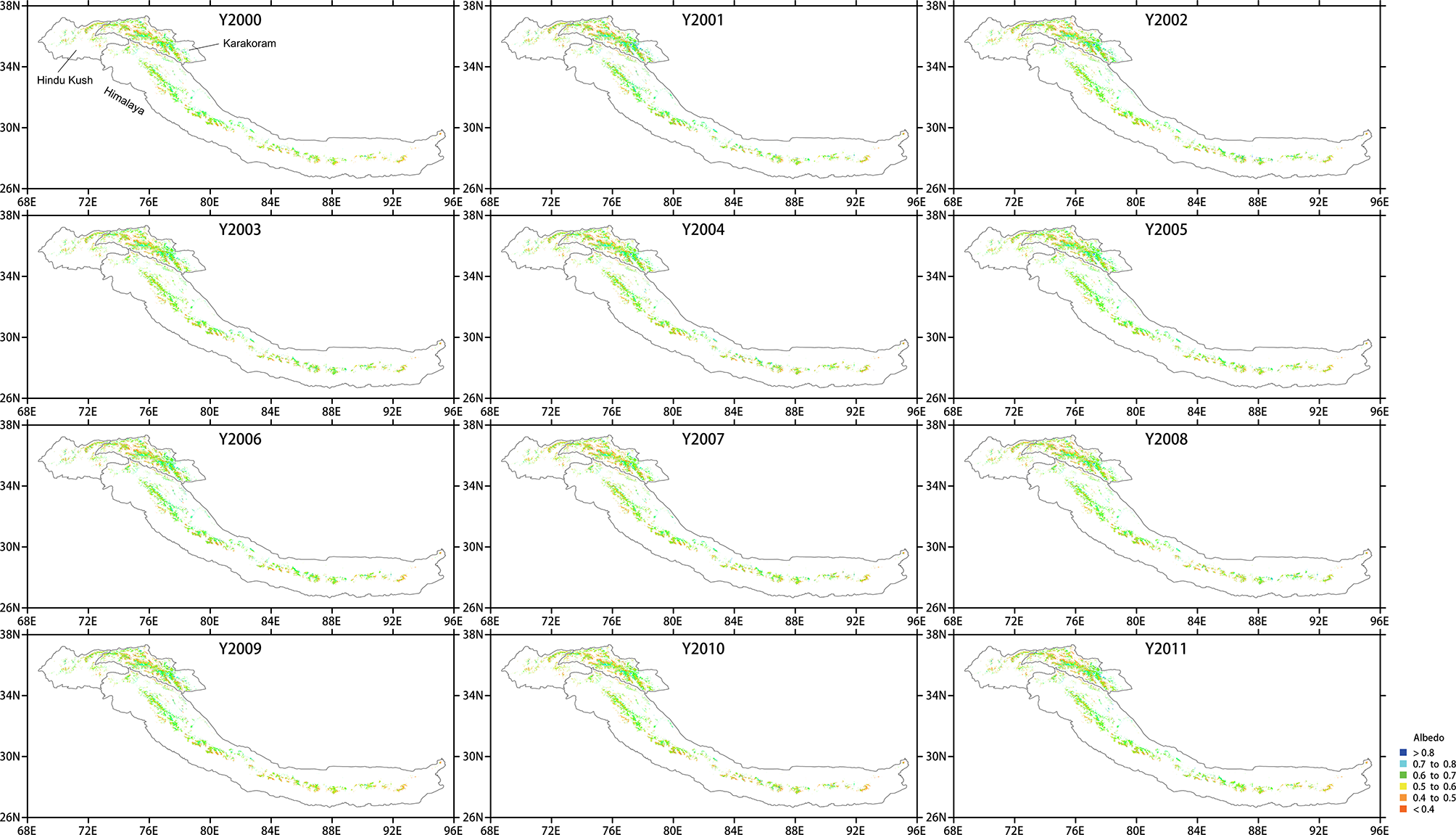

Supplement: S4 Fig — (TIF) [file pone.0126235.s004.tif]

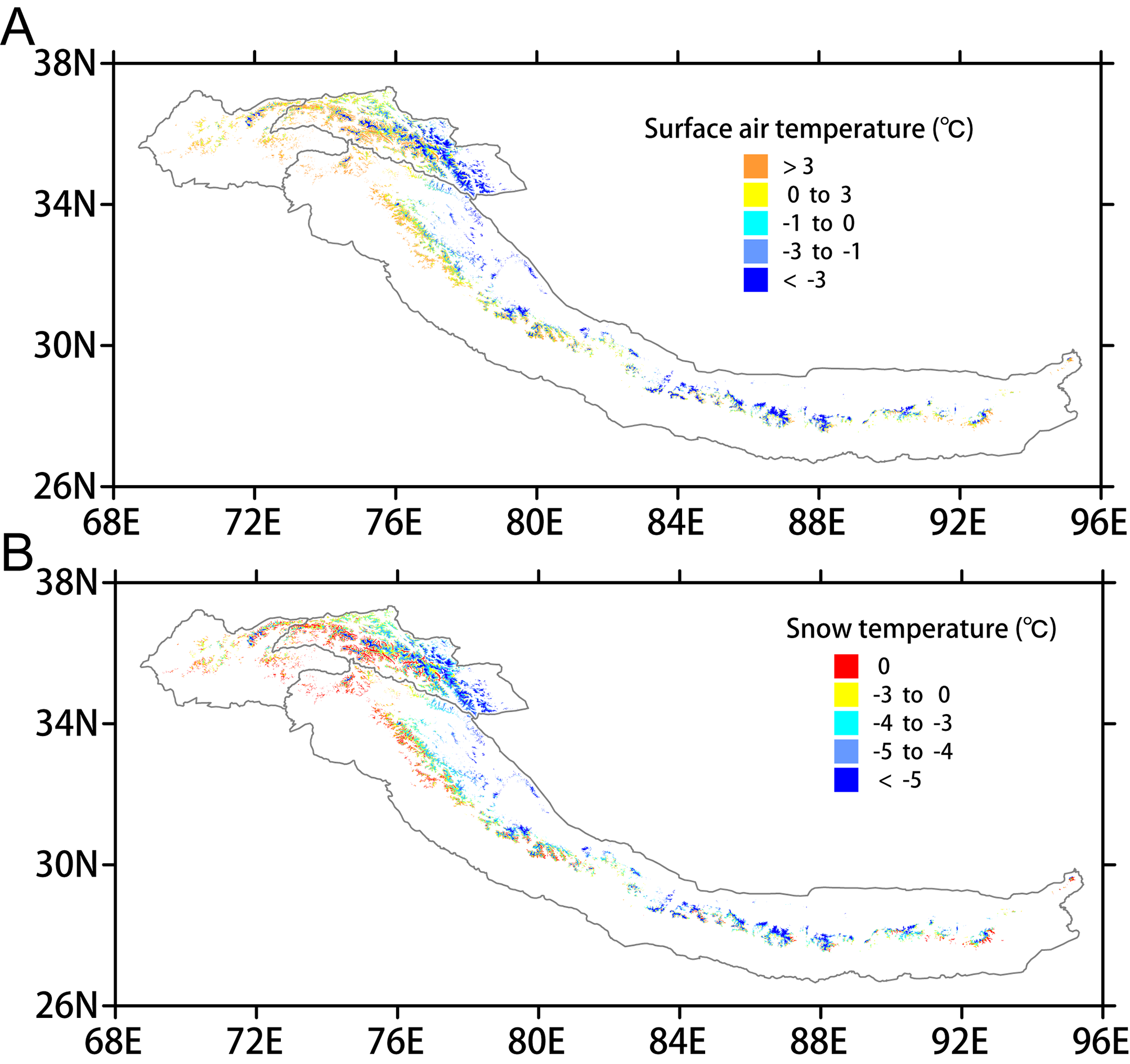

Supplement: S5 Fig — (TIF) [file pone.0126235.s005.tif]

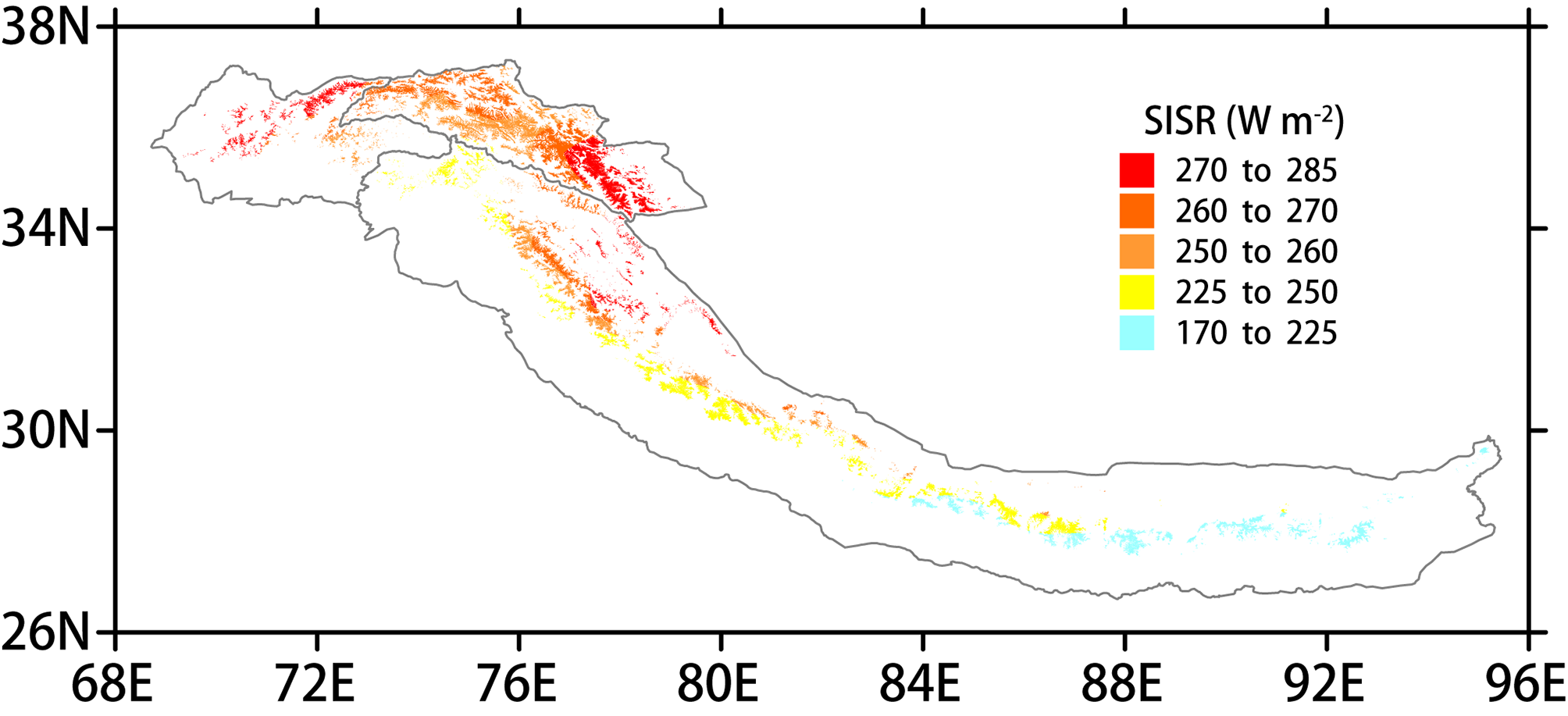

Supplement: S6 Fig — (TIF) [file pone.0126235.s006.tif]
